# A three-dimensional computational study of critical pressures of dissection propagation in the aorta

**DOI:** 10.1007/s10237-025-01991-2

**Published:** 2025-07-31

**Authors:** Sathish Kumar Marimuthu, Giulia Luraghi, Craig Maclean, Robbie Brodie, Francesco Migliavacca, Sean McGinty, Nicholas A. Hill

**Affiliations:** 1 School of Mathematics and Statistics, University of Glasgow, Glasgow, G12 8QQ UK; 2 Division of Biomedical Engineering, University of Glasgow, Glasgow, G12 8QQ UK; 3 Computational Biomechanics Laboratory – LaBS, Department of Chemistry, Materials and Chemical Engineering ‘Giulio Natta’, Politecnico di Milano, Piazza L. da Vinci 32, 20133 Milan, Italy; 4 R&D, Terumo Aortic, New Mains Avenue, Renfrewshire, Inchinnan, PA4 9RR UK; 5 Fondazione IRCCS Ca’ Granda Ospedale Maggiore Policlinico, Milan, Italy

**Keywords:** Aortic dissection, Tear propagation, Finite element method (FEM), Extended finite element method (XFEM), Damage mechanics

## Abstract

Aortic dissection is a life-threatening disease with high mortality rates. The degradation of the layers of the aorta wall causes tears, which then propagate further due to high-pressure blood penetrating the vessel wall, creating a false lumen. The intimal flap separating the true and false lumen can either bulge inwards constricting the true lumen’s blood flow or bulge outwards leading to catastrophic rupture and internal bleeding. Therefore, to understand the role of critical pressure on tear propagation, a computational study of the initiation and propagation of tears of various sizes and at multiple depths and locations in three-dimensional aortas was conducted. Tears were modelled using the extended finite element method, and the wall of the aortas is an anisotropic hyperelastic material. Blood-pressure-loaded aorta geometries were obtained from the corresponding unloaded geometries using an iterative procedure to match the in vivo geometries. Pressure-driven tear initiation and propagation were studied. Our results show that when the tear surface’s normal is perpendicular to the blood flow, the critical pressure required to cause further propagation is higher for the shorter and deeper tears and reduces when the initial tear size increases. When the normal is parallel to the blood flow, the difference in critical pressure with an increase in tear depth is small and is more likely to propagate transversely. Also, the critical pressure decreases with an increase in the diameter of the aorta for all the tear orientations. This study concludes that tear size, depth inside the medial layer and the diameter of the aorta near the tear location are critical parameters in assessing the risk of further propagation.

## Introduction

**Fig. 1:**
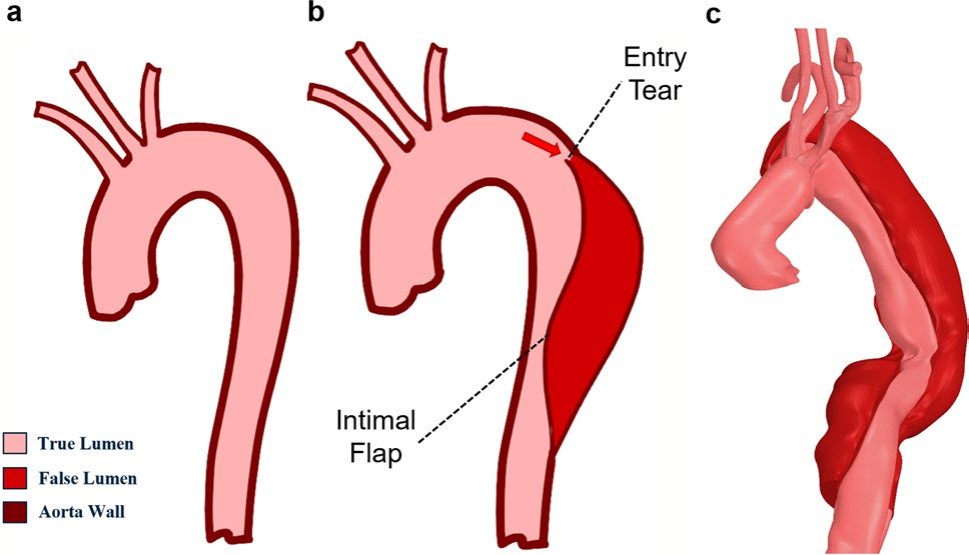
Illustrations of a normal aorta (**a**), dissected aorta (**b**) and dissected patient aorta (**c**) showing the true lumen and false lumen created due to disease progression. The red arrow shows an entry tear through which the blood enters the dissected aorta wall and the false lumen

Aortic dissection is a serious cardiovascular disease with a high mortality rate. Prompt medical attention is required, with the risk of death increasing with time from the onset of the dissection. The occurrence of aortic dissection is initiated with an injury or degradation of the intimal layer of the supposedly weakened aorta wall (Gültekin et al. 2019; Sherifova and Holzapfel 2019). The arterial wall degeneration causes the reduction in cohesiveness between the layers leading to delamination (Khan and Nair 2002). Then, the tear tends to propagate further into the medial layer and the blood from the true lumen leaking into the tear creates an additional cavity known as the false lumen (Fig 1). Under further loading from the additional blood flow into the false lumen, the tear is prone to delaminate the layers and cause the bulging of the false lumen (Mikich 2003). The bulging of the false lumen internally towards the true lumen may constrict the blood flow in the true lumen and even cause re-entry tears distally (Criado 2011; Gasser and Holzapfel 2006; Gawinecka et al. 2017). The progressive bulging of the false lumen can also lead to rupture of the aorta wall in the event of damage to the adventitial layer and cause internal haemorrhages, which can be fatal (Gawinecka et al. 2017).

Aortic dissection incidences are evidenced in up to 30 to 40 cases per million people annually (Criado 2011; Khan and Nair 2002; Kurz et al. 2017). Anatomically, dissections occurring on the aorta wall proximal and distal to the left subclavian artery are known as proximal aortic dissections and distal aortic dissections, respectively (Khan and Nair 2002). Crucial factors correlated with the dissection are hypertension, old age, and large haemodynamic forces at the critical locations, leading to the degradation of the aorta wall (Criado 2011; Robicsek and Thubrikar 1994; Sommer et al. 2016). Patients with connective tissue disorders, such as Marfan’s syndrome or other hereditary defects, are also found to be at risk of forming dissection (Robicsek and Thubrikar 1994). Mean and maximum systolic pressure influence the initiation of dissection, and pulse pressure and cycle frequency affect dissection propagation (Rajagopal et al. 2007).

Constitutive modelling of the layer-specific collagen fibre-reinforced anisotropic structure of the large arteries was pioneered by Holzapfel et al. (2000) and then by Gasser and Holzapfel (2006), accounting for the fibre dispersions around the mean fibre directions. Then, the initial studies on the remodelling of the aorta in aneurysmal cases and aortic dissection propagation were conducted by Watton and Hill (2009), Watton et al. (2004), and Gasser and Holzapfel (2006) respectively, which led to advanced developments in the studies on aortic disease progression. Gasser and Holzapfel (2006) modelled the dissection propagation numerically using the extended finite element method (XFEM). Sommer et al. (2008) conducted direct tension and peeling tests, while Sommer et al. (2016) conducted triaxial shear and uniaxial tensile tests on strips cut from the dissected aorta specimens and evaluated the ultimate failure stresses. Wang et al. (2016) and Wang et al. (2018) studied dissection propagation in residually stressed axisymmetric tissue specimens, and Brunet et al. (2021) and Han et al. (2023) studied propagation in residually stressed 3D cylinders for a range of tear sizes using XFEM. Furthermore, Gültekin et al. (2019) used crack phase-field methods to study propagation in residually stressed cylindrical aortas and the application of such advanced tear propagation models for 3D aorta geometries is yet to be conducted.

To the authors’ knowledge, all the previous literature conducted dissection propagation studies on simplified 2D or 3D (cylindrical) geometries and this is the first numerical study of dissection propagation in 3D aorta geometries derived from CT scans with physiological pressure loads. Fitzgibbon et al. (2020) used advanced cohesive zone methods (CZM)-based fracture models to study the dissection risk in 3D patient-specific aortas. However, their study did not include any tear propagation and the techniques developed in this study could be coupled with such advanced models in future studies to model arbitrary tear propagation in 3D patient-specific aorta geometries. In this study, we considered the effects of the pre-loaded aorta’s different (predefined) tear sizes and diameters on further tear propagation. The tear sizes range from 3 mm to 12 mm at three depths inside the aorta wall. Typical locations of aortic dissection occurrences are the right lateral wall of the ascending thoracic aorta opposite the pulmonary artery (Gawinecka et al. 2017), near the ligamentum arteriosum and left subclavian artery (Khan and Nair 2002) and in the descending aorta in the case of re-entry tear formation. Hence, three locations for the tears: ascending aorta (near the right lateral wall and opposite to the pulmonary artery), immediately after the left subclavian artery (near ligamentum arteriosum) and further down in the descending aorta, were studied.

**Fig. 2:**
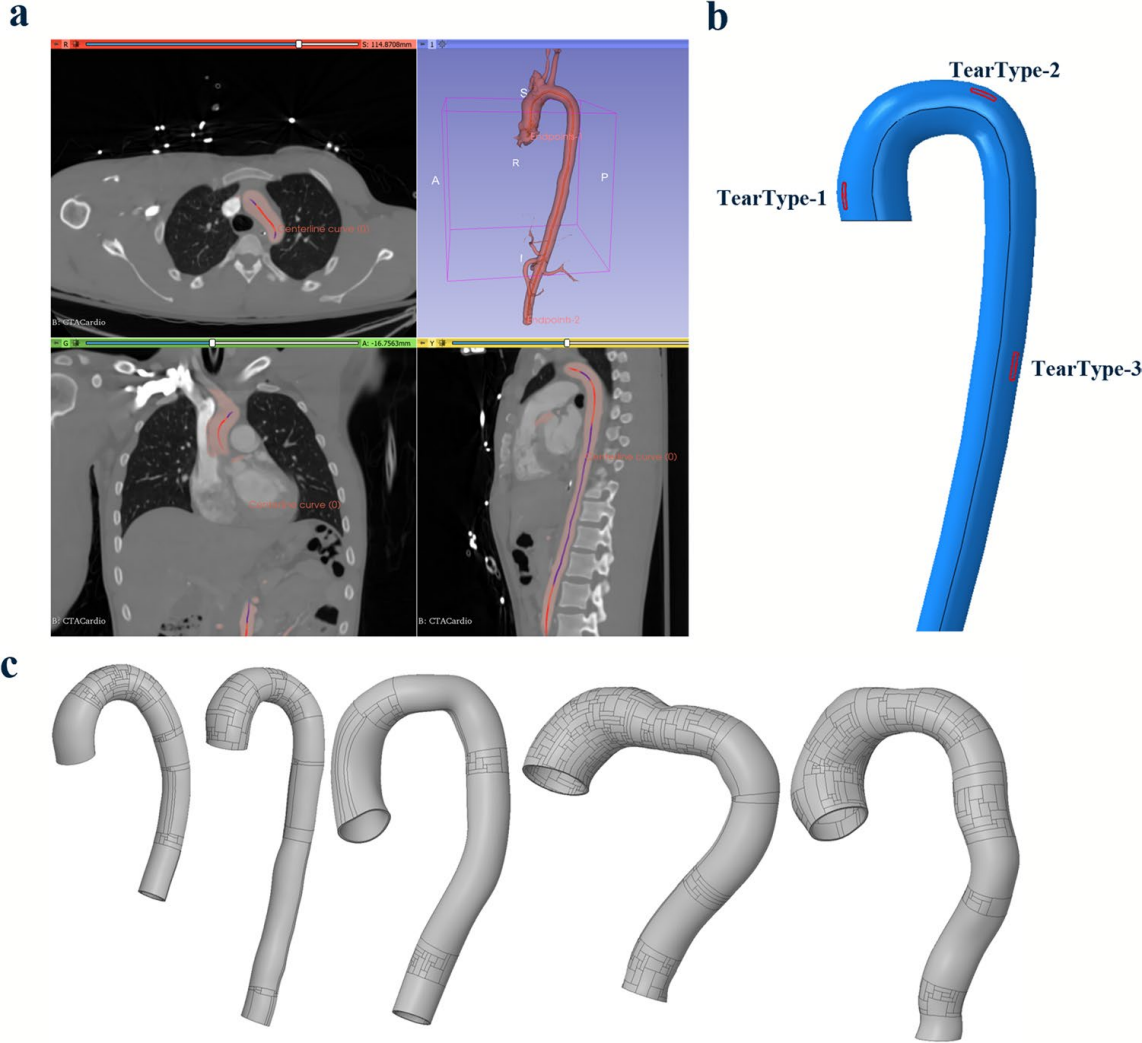
**a** Segmentation and centreline extraction to generate a 3D aorta geometry from a sample CT, **b** The three dissection occurrence locations at which we considered our tear surfaces and **c** five patient aorta geometries used for the study. The tears shown here are in $\theta z$ orientations and are mentioned as Tear types 1, 2 and 3 based on the location. Note: The patches on the aorta geometries in **c** are not initial tears and they only appear in the visualisation of computer-aided design (CAD) files. These patches are smaller faces which capture the curvature of the geometries properly and are the result of conversion between a stereolithography (STL) file to a parametric CAD file

## Methods

### Geometry and boundary conditions

Five semi-idealised three-dimensional (3D) aorta geometries were considered in this study (Fig 2c). Two aortas were generated in Solidworks (Dassault Systemes Solidworks Corp., MA, USA) using sample (Fig 2a) anonymized computerised tomography (CT) scans (Fedorov et al. 2012; Karim et al. 2018), and three other geometries were obtained from Terumo Aortic. Three healthy aortas and two diseased aortas with aneurysmal ascending parts were considered for the study. The inclusion of branching arteries is necessary to model the propagation of the dissection. However, this study only focuses on estimating critical pressure for the onset of dissection. Therefore, the initial tears were positioned away from the left subclavian artery location, and the subordinate or branching arteries were excluded, assuming they do not significantly influence the critical pressure for the onset of dissection. The intimal layer was neglected due to its negligible mechanical contribution to the strength of the aorta (Gültekin et al. 2019). The thickness of the media and adventitia were assumed to be 1.5 mm and 0.5 mm, respectively. The longitudinal and circumferential displacements were restricted for the two ends of the aorta, and only radial displacements were permitted. Mesh-independence studies were conducted for the aorta geometries and meshed with hexahedral elements, with reduced integration and enhanced hourglass controls in the implicit solver Abaqus Standard 2021 (ABAQUS 2014).

**Fig. 3:**
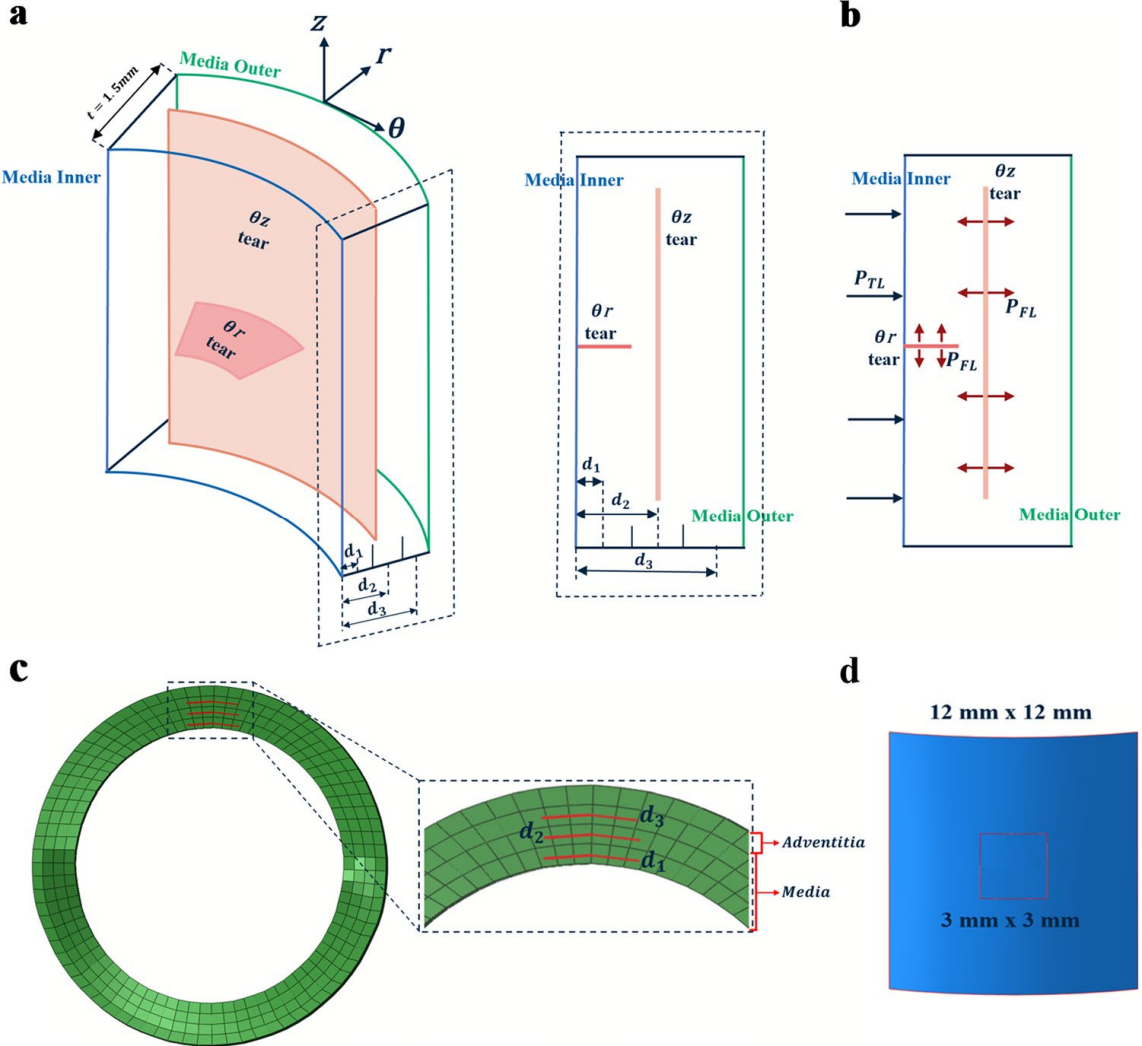
**a** Illustration of the setup of two orientations of the tears inside the medial layer, **b** Pressure load on true and false lumens, **c** Tears at three different depths (0.25 mm, 0.75 mm, 1.25 mm) inside the aorta media and **d** the range of initial tear sizes

The tear surfaces were designed using the cylindrical polar coordinate system in which $\theta$, *r*, and *z* represent the circumferential, radial and longitudinal (or axial) directions in the current configuration. $\theta z$ tears (surfaces with unit normal parallel to the radial direction, *r* ) were studied in all five aortas and $\theta r$ tears (surfaces normal to axial (blood flow) direction, *z*) in the three healthy aortas (Fig 3), because tears connecting the true and false lumen were significantly necessary for the initial tear formation. The $\theta r$ tear orientation represents the initial phase of the tear occurrence, and $\theta z$ tear orientation represents the propagation phase. The definition of $\theta r$ tears in the other two aortas was hampered due to their overly bulged ascending parts. Additionally, $\theta r$ tears were initially tested for the three healthy aorta models, and it was found that tear size and depth did not affect the critical pressure. Moreover, the $\theta r$ tear represents the initial tear formation due to the mechanobiological degradation of the aorta wall. This study mainly focused on the pressure-induced delamination of the wall and did not focus on the aneurysm leading to dissection or rupture. Hence, the $\theta r$ tears were only simulated for the three healthy cases. The sizes ($\theta \times z$) for the $\theta z$ tears range from 3 mm $\times$ 3 mm to 12 mm $\times$ 12 mm (Fig 3d), and sizes ($\theta$) for the $\theta r$ tears range from 3 to 12 mm.

**Fig. 4:**
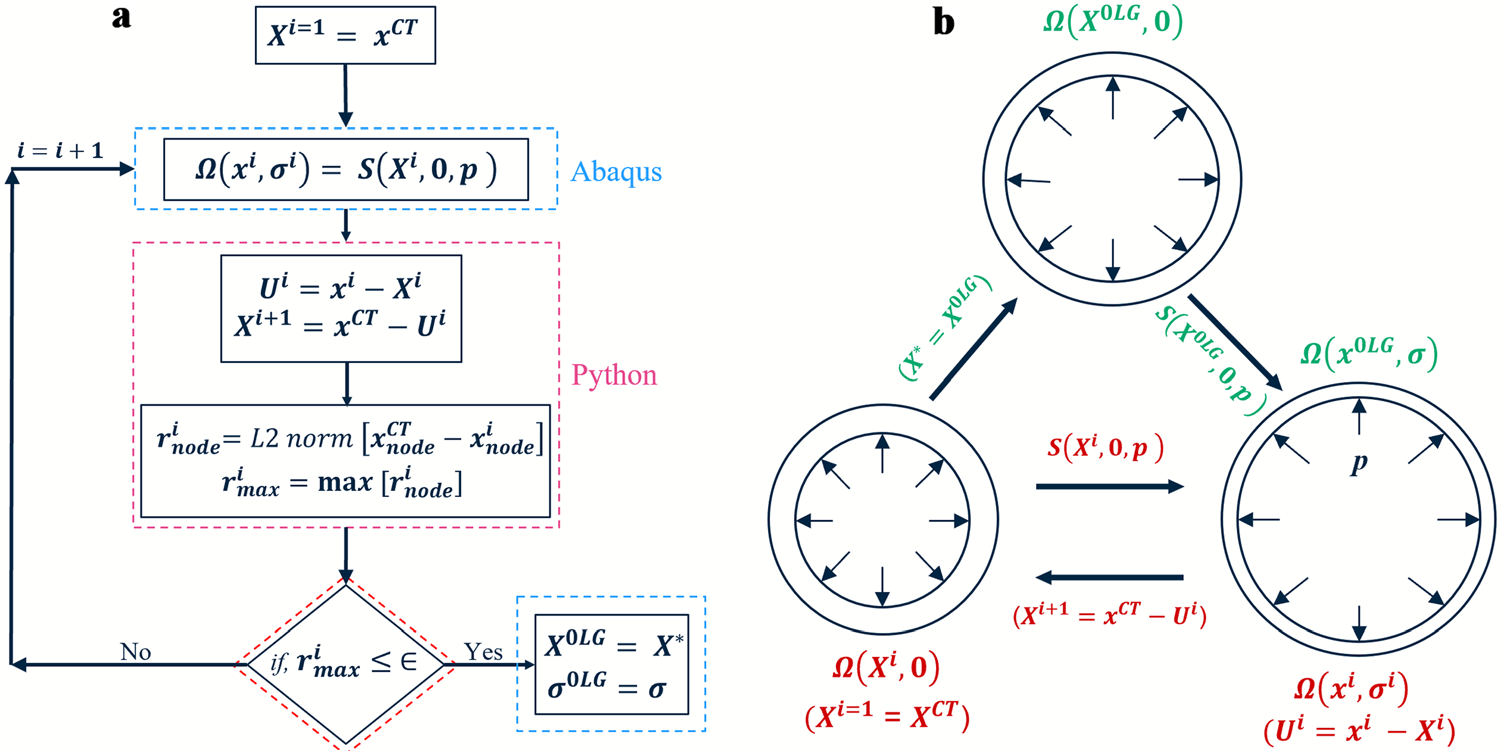
Forward-and-backward analysis algorithm for obtaining zero-load state geometry from CT scan image (Bols et al. 2013). **a** The iterative algorithm and **b** unloaded and loaded aortas at different stages of the procedure. $\textbf{S}(\textbf{X}^i,0,p)$ depicts the iterations and $\textbf{S}(\textbf{X}^{0LG},0,p)$ depicts the simulation with final zero-load geometry (0*LG*). $\textbf{X}$ and $\textbf{x}$ are the position vectors of a point, and $\mathbf {\sigma }^{0LG}$ and $\mathbf {\sigma }$ are the Cauchy stresses in the reference ($\Omega (\textbf{X})$) and current ($\Omega (\textbf{x},\mathbf {\sigma })$) configurations, respectively. $\textbf{U}$, *p* and $\textbf{r}$ are displacement, pressure and residue vector from the L2-norm

The CT scan data represents the in vivo aorta subjected to loading due to blood pressure and residual stresses. Ignoring these residual stresses will lead to unphysiological results. In order to obtain the zero-load geometry in the reference configuration from the in vivo geometry, we employed a ‘forward-and-backwards’ analysis (Bols et al. 2013; Riveros et al. 2013). Figure 4 displays the flowcharts of the iterative procedures to obtain (i) the zero-load aorta from the in vivo geometry and (ii) the loaded aorta from the zero-load aorta geometry. In the iterative procedure (Fig. 4), the in vivo geometry generated using the CT scan data ($\textbf{x}^{CT}$) was used as an initial geometry ($\textbf{X}^{0}$). In the forward analysis step, a diastolic pressure load of 80 mmHg (p) was applied to the inner wall of the aorta (Bols et al. 2013), which has zero stresses. The simulation was carried out in the implicit Abaqus Standard 2021 (Dassault Systemes Simulia Corp., RI, USA) solver, and the displacements of the nodes for the inflated intermediate geometry were measured ($\textbf{U}^{i+1}$). Then, in the backward analysis step, the intermediate zero-load geometry ($\textbf{X}^{i+1}$) was then obtained by subtracting the calculated nodal displacements from the initial in vivo geometry ($\textbf{x}^{CT}$) using Python scripts. Summarising, nodal displacements while inflating the initial geometry were recorded, and then these were subtracted from the initial geometry to obtain the zero-load geometry. The nodal coordinates of the aorta were updated in the input file to initialise the geometry for the next iteration. The forward-and-backwards analysis was iterated until the zero-load geometry matched the in vivo geometry when inflated to diastolic blood pressure. The corresponding final zero-load geometry ($\textbf{X}^{*}$) was pressurised to obtain the loaded aorta with the stresses ($\mathbf {\sigma }^*$), matching the in vivo conditions.

The final geometry corresponds to the aorta in the loaded configuration, which incorporates the stresses due to loading. The iteration procedure was stopped if the residue ($\mathbf {r_{max}}^{i}$), which was the maximum L2-norm, was less than or equal to a critical value of 0.01 mm (less than the CT spatial resolution of 0.5 mm, Lin and Alessio (2009)). Coordinates of the final zero-load geometry were updated in the input file, and the tear simulation was carried out in two steps: (1) inflation of the zero-stress geometry with diastolic blood pressure to obtain the loaded aorta, and (2) further pressurisation of the false and true lumen to investigate the tear initiation and propagation. The XFEM tear growth was enabled in the false lumen pressurisation step.

### Constitutive and equilibrium equations

The media and adventitia layers of the zero-stress aorta geometry were modelled using the incompressible anisotropic hyperelastic GOH (Gasser-Ogden-Holzapfel) material (Gasser et al. 2006). $\mathbf {\chi }(\textbf{X},t)$ maps the material points in the reference configuration ($\textbf{X}$) to corresponding spatial points in the deformed or current configuration $\textbf{x}= \mathbf {\chi }(\textbf{X},t)$. The local deformation is characterised by the deformation gradient, $\textbf{F}(\textbf{X},t) = \frac{\partial \mathbf {\chi }(\textbf{X},t)}{\partial \textbf{X}}$, at a material point and the right Cauchy–Green strain tensor (the deformed squared length of a material vector gives $d\textbf{x}^2=d\textbf{X}^T \cdot \textbf{C}d\textbf{X}$) is defined to be $\textbf{C}=\textbf{F}^T\textbf{F}$. The distortional part of the deformation gradient is $\bar{\textbf{F}} = J^{-\frac{1}{3}} \textbf{F}$ where $J(\textbf{X},t) = \text {det}\textbf{F}(\textbf{X},t)$ is the Jacobian or volume ratio. The distortional part of the right Cauchy–Green strain tensor is $\bar{\textbf{C}} = J^{-\frac{1}{3}} \textbf{C} = \bar{\textbf{F}}^T\bar{\textbf{F}}$. The strain-energy function is decoupled into the dilatational and the distortional parts for computational efficiency, and the GOH strain-energy potential (Gasser et al. 2006) is

1 $$\begin{aligned} & \Psi = \frac{1}{D}\left( \frac{(J^2-1)}{2} - \ln J\right) + C_{10} (\bar{I}_1 - 3) + \frac{k_1}{2 k_2} \sum _{\alpha =1}^{N=2} \left( \exp (k_2 {\langle \bar{E}_\alpha \rangle }^2) - 1 \right) \, \end{aligned}$$

2 $$\begin{aligned} & \bar{E}_{\alpha =4,6} = \kappa (\bar{I}_1 - 3) + (1 - 3 \kappa )(\bar{I}_{4\alpha } - 1) \, \end{aligned}$$

3 $$\begin{aligned} & \langle \bar{E}_{\alpha } \rangle = \frac{1}{2} (|\bar{E}_{\alpha }| + \bar{E}_{\alpha }). \end{aligned}$$

where $\Psi$ is the strain-energy per unit reference volume, $\bar{E}_{\alpha }$ is a strain-like parameter, $C_{10}$, *D*, $k_1$, $k_2$, and $\kappa$ are material parameters, *N* is the number of families of fibres, $\bar{I}_1$ is the first invariant of $\bar{\textbf{C}}$ ($\bar{I}_1 = \text {tr} \bar{\mathbf{C}}$) and $\bar{I}_{4i} = \mathbf {\bar{C}}: \textbf{A}_{\alpha } \otimes \textbf{A}_{\alpha }$ are pseudo-invariants of $\bar{\textbf{C}}$ and the fibre directions $\textbf{A}_{\alpha }$. The material parameter values for the media and adventitia layers of the aorta are shown in Table. 1. The ground matrix is assumed to be an anisotropic neo-Hookean material, and the anisotropic contribution appears only if $\bar{E}_{\alpha }>0$, as the fibres can only support tension and not compression. This assumption can lead to discontinuous or non-smooth behaviour during the initial recruitment of the collagen fibres. However, the minimum transmural pressure occurs at the end of diastole, and the application of an 80 mmHg load at the start ensures that no collagen fibres are in compression.

**Fig. 5:**
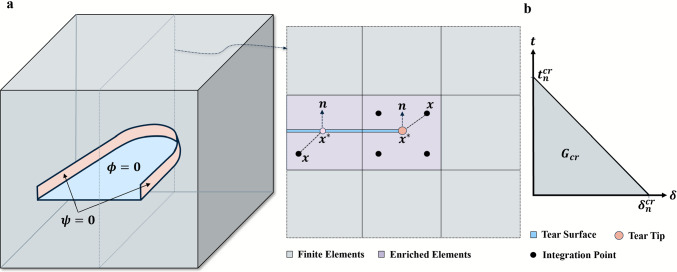
**a** Illustrations of XFEM tear definition inside a continuum body with conventional finite elements and enriched XFEM elements in the vicinity of the tear (adapted from ABAQUS (2014)). A signed level-set distance function $\phi$ describes the tear surface, and the tear front is defined by the intersection between $\phi$ and another signed distance function $\psi$ describing an orthogonal surface. **b** Linear-traction separation law that governs the tear evolution after the initiation

### eXtended finite element method (XFEM) and damage model

Conventional finite element methods (FEM) (Zienkiewicz et al. 2014) require mesh refinement near the tear surfaces as the tear front is not able to pass through the elements, increasing the complexity. However, XFEM (Moës et al. 1999) uses additional enrichment terms with the usual displacement functions to capture the tear initiation and propagation, and the tear front can propagate through the elements. Extension of the conventional finite element method based on the concept of partition of unity and the approximation for the displacement vector function (ABAQUS 2014) is

4 $$\begin{aligned} \textbf{u} = \sum _{I=1}^N N_I (x)[\textbf{u}_I + H(x) \textbf{a}_I + \sum _{\alpha = 1}^4 F_\alpha (x) \textbf{b}_I^\alpha ] \ . \end{aligned}$$

where $N_I (x)$ are the usual nodal shape functions, $\textbf{u}_I$ is the usual nodal displacement vector (applicable to all elements), $\textbf{a}_I$ is the degrees of freedom vector of the enriched elements where the tear surface is present and $\textbf{b}_I^\alpha$ is the degree of freedom vector of the enriched elements where the tear tip is present. *H*(*x*) is a discontinuous jump function across tear surfaces and $F_\alpha (x)$ is the asymptotic tear tip function. The jump in displacement is governed by a discontinuous jump function, which is given as

$$\begin{aligned} H(x) = {\left\{ \begin{array}{ll} \quad 1 & \text {if} \ \left( \textbf{x} - \textbf{x}^*\right) \cdot \textbf{n}\ge 0 \, \\ -1 & \text {otherwise} \, \end{array}\right. } \end{aligned}$$

where $\textbf{x}$ is the sample Gauss point (integration points defined by the Gaussian quadrature in FEM), $\textbf{x}^*$ is the point on the tear closest to the sample Gauss point ($\textbf{x}$), and $\textbf{n}$ is the unit outward normal to the tear surface at $\textbf{x}^*$ (Fig. 5a). The terms associated with the nodes near the tear tip in Abaqus (ABAQUS 2014) are ignored for the propagating tears. To avoid modelling the stress singularity near the tear tip, the tear is assumed to propagate across an entire element (ABAQUS 2014). The level-set method uses two orthogonal signed distance functions to describe the discontinuous geometry or the tear surface. Signed level-set distance function $\phi$ describes the tear surface, $\psi$ describes the orthogonal surface, and the intersection gives the tear front (Fig. 5a). The initial tear surface (false lumen surface) is defined in the assembly without any need for meshing as it is placed in the interpolation of the displacement function $\textbf{u}$. Unlike the element-based cohesive segments method, by using surface-based cohesive segments methods and XFEM, the tear can propagate along solution-dependent arbitrary paths.

The onset of the tear initiation is assumed to occur when the maximum of the ratios (*f*) between the traction stress vector, $\textbf{t} (t_n,t_s,t_t)$ and the associated peak critical value, $t^{cr}=(t_n^{cr},t_s^{cr},t_t^{cr})^T$, equals one. Here, *n*, *s*, *t* denote the normal and the other two perpendicular (shear) directions. As purely compressive stresses do not contribute to the tear initiation, the Macaulay bracket $\langle t_n \rangle = \frac{1}{2} \left\{ \langle |t_n |\rangle + \langle t_n \rangle \right\}$ denotes that only tensile stresses cause the tear initiation:

5 $$\begin{aligned} f= & \max \left\{ \frac{\langle t_n \rangle }{t_n^{cr}}, \frac{t_s}{t_s^{cr}}, \frac{t_t}{t_t^{cr}} \right\} \ , \end{aligned}$$

6 $$\begin{aligned} \textbf{t}= & \begin{bmatrix} t_n\\ t_s\\ t_t\\ \end{bmatrix} = \begin{bmatrix} K_{nn}& 0& 0\\ 0& K_{ss}& 0\\ 0& 0& K_{tt}\\ \end{bmatrix} \begin{bmatrix} \delta _n\\ \delta _s\\ \delta _t\\ \end{bmatrix} = \mathbf {K \delta } \ . \end{aligned}$$

The evolution of tear propagation is governed by a linear traction-separation law (Fig. 5b), which relates the surface tractions and the jumps in displacements. The elastic constitutive matrix relates normal and shear tractions ($\textbf{t} (t_n,t_s,t_t)$) to the normal and shear separations ($\mathbf {\delta } (\delta _n,\delta _s,\delta _t )$) of a torn element (ABAQUS 2014). The stiffness terms $\textbf{K}$ and traction-separation behaviour are calculated by specifying the elastic properties of the material in an enriched region (ABAQUS 2014). The critical values for nominal stresses and fracture energy based on the tests of (Sommer et al. 2008, 2016) are shown in Table. 1.

**Table 1:** Parameters for the anisotropic hyperelastic material properties of the aorta and the fracture criteria for modelling tear initiation and propagation (Brunet et al. 2021; Han et al. 2023; Sommer et al. 2016)

Aorta	$C_{10}$	$k_{1}$	$k_{2}$	$\kappa$	$\theta$	*T*	$\sigma _{nom}^{max}$ (kPa)			*G*
	(kPa)	(kPa)			$(^\circ )$	(mm)	${t_n}^{cr}$	${t_s}^{cr}$	${t_t}^{cr}$	(*N*/*m*)
Media	16	90	19	0.12	41.6	1.5	131	97	120	50
Adventitia	13.5	370	15.2	0.25	54.8	0.5	131	97	120	50

### Tear modelling

The tear surfaces inside the aorta media wall were modelled with the XFEM tear growth method in Abaqus/Standard 2021 (Dassault Systemes Simulia Corp., RI, USA). Tear surfaces were designed such that when the zero-load geometry of the aorta is pressurised, the surfaces reach the expected final sizes. As the tears are expressed in terms of the relative distance between tear surfaces and nodes of the enriched elements, it is convenient to define them in the centre of the elements without cutting the element edges. Figures 3a-c illustrate the setup of the initial false lumen surfaces inside the wall of the aorta media and the pressurisation of the true and false lumen surfaces.

**Fig. 6:**
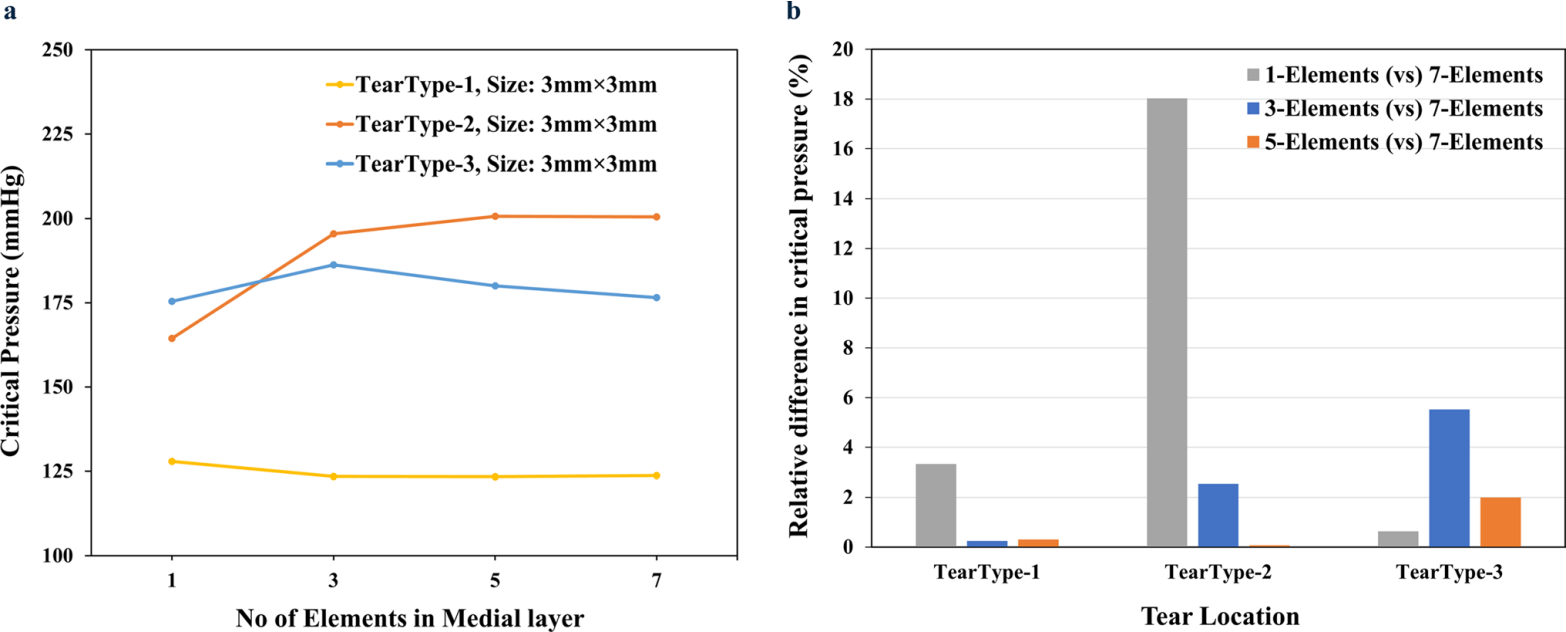
Mesh-independence test for choosing an optimal number of elements in the medial layer by simulating the $\theta z$ tear propagation using simultaneous true and false lumen pressurisation. **a** Critical pressure versus the number of elements plot and **b** the comparison between the 7-element cases with other numbers of elements across the aorta wall of the first specimen (S1). Tear types 1, 2 and 3 denote tears at the ascending, arch and descending aorta regions

To conduct mesh independence analysis, the medial layer of the first aorta specimen (S1) was discretised with either 1, 3, 5 or 7 (baseline) hexahedral elements through its thickness and a 3 mm $\times$ 3 mm tear was defined at the middle of the medial layer for all the cases. Results indicate that choosing 3 elements along the medial thickness results in less than 6% differences in critical pressure compared to the 7-element case (Fig 6). Taking account of the computation time with an increase in the number of elements, 3 elements through the thickness of the media was considered for all the subsequent simulations. The adventitial layer was modelled as one layer of elements, and the intimal layer was ignored due to its negligible effect on mechanical strength. Also, the aorta wall thickness was assumed to be uniform for simplicity. The tears were defined at three different depths ($d_1$, $d_2$ and $d_3$) at the centre of the three elements along the wall (Fig 3c). Both the $\theta z$ and $\theta r$ tear surfaces of sizes ranging from 3 to 12 mm were defined at the three different locations in the aorta (Fig 2b). The arc length of the $\theta r$ tear ranged from 3 to 12 mm. The size in *r* direction was denoted by the predefined depth (Fig 3a) and the whole radial length of the $\theta r$ tear was unnecessary for the initial crack definition.

The inner surface of the true lumen was pressurised with the diastolic blood pressure of 80 mmHg to obtain the pre-stressed aortas. Tear propagation was switched off while obtaining the pre-loaded aorta geometry. In the second step, the inner surface of the true and false lumen cavities was pressurised from 80 mmHg baseline, and the tear propagation was switched on. As the blood flowing into the false lumen is the key factor in further tear propagation, the false lumen surfaces were also pressurised to the 80 mmHg diastolic pressure at the start of the second step. Bäumler et al. (2020) state that the true lumen pressure is approximately 5 mmHg higher than the false lumen pressure in the initial phase of the cardiac cycle (peak pressure zone), so the differences between the true lumen and false lumen pressures are relatively small. We did not consider flow, and there are no re-entry tears, so it is a reasonable approximation to assume the pressures are the same in true and false lumen. XFEM tear growth interaction in Abaqus was used to activate and deactivate the tear initiation. The false lumen cavity surfaces were defined with hard contact normal behaviour and friction formulation tangential behaviour to avoid penetration of the two contacting surfaces. All the simulations run in an implicit Abaqus Standard solver with 12 cores. It took approximately 30 min in a Linux server with 104 logical cores and 395GB RAM capacity. As the tear propagation is rapid and unstable, the simulations were stopped at the onset of further tear propagation. Tear propagation takes a large number of iterations and increases the computational time tremendously. The critical pressure applied on the false lumen surfaces and the inner surfaces of the true lumen of the aorta was calculated.

## Results

**Fig. 7:**
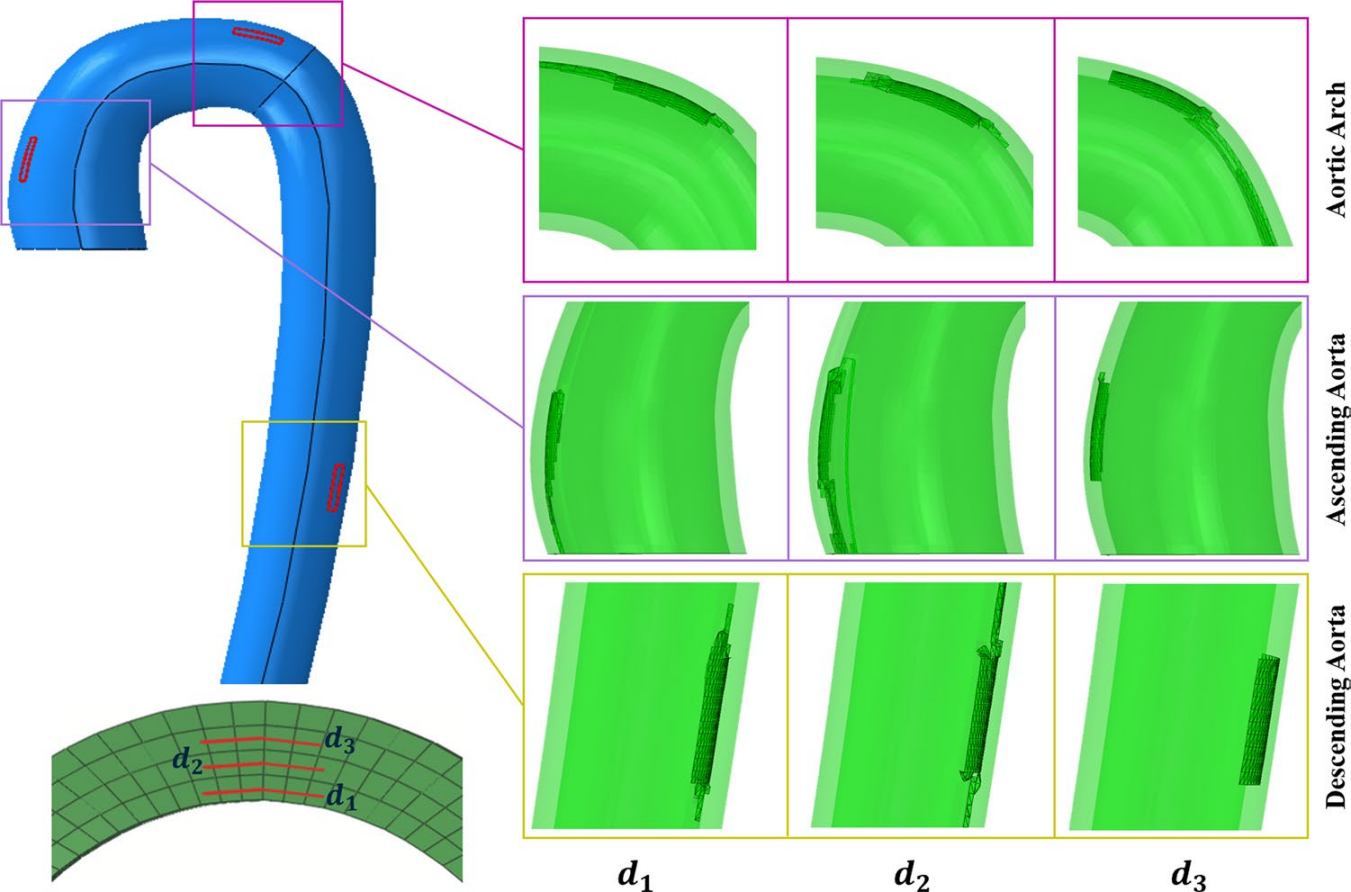
Propagation of 12 mm $\times$ 12 mm tears defined at three different depths ($d_1$) and three different regions (ascending aorta, aortic arch and descending aorta). Red-coloured rectangles on the aorta (blue colour in the reference configuration) in three locations are the initial predefined 3D tear surfaces defined inside the medial layer. The aorta (green colour in the current configuration) is sliced near the tears in the aortic arch, ascending and descending aorta regions to show the locally propagated tears (torn XFEM elements are shown in a dark shade, inside the green aorta regions)

The propagation of tears of different sizes ranging from 3 to 12 mm was studied at three different locations and depths inside the aorta wall. The corresponding critical pressures are plotted against the depth inside the aorta wall. Due to the limited sample size of only five patient geometries, the data is insufficient for statistical analysis. The influences of the depth of tear inside the media, size of the tear cavity and orientations of the initial tear surfaces on the critical pressure to cause further tear propagation were studied. Since the branching arteries were removed for simplification, the diameters at the arch do not exactly match as the holes in the place of the branching arteries were closed for simplifying the meshing procedure. Even though we defined the tear only after the left subclavian artery, the behaviour was found to be similar between the arch and descending aorta. Therefore, the aorta diameters at the centre of the tears located at the arch and descending parts of the five aortas used in this study are shown in Table 2. Since the focus here is on the critical pressure at the onset of propagation, all the simulations were stopped immediately after the propagation was initiated to reduce the computation time and space, with the exception of a few representative examples in which 12 mm $\times$ 12 mm tears with $\theta z$ orientation that were allowed to propagate further inside the medial layer when the lumens were simultaneously pressurised ($P_{TL} = P_{FL}$) are plotted in Fig. 7.

**Table 2:** Aorta diameters at the centre of the tears, located at the ascending and descending parts of five aorta samples. Ascending aorta diameters were larger than the descending aorta

Aorta sample	Aorta diameter at the tear location (mm)	
	Ascending aorta	Descending aorta
Aorta - 1	26.107	16.444
Aorta - 2	23.014	15.681
Aorta - 3	29.005	24.147
Aorta - 4	40.456	26.764
Aorta - 5	41.381	27.096

These values were taken from the CT scan geometries, and we assume the scans were taken during the diastolic phase

**Fig. 8:**
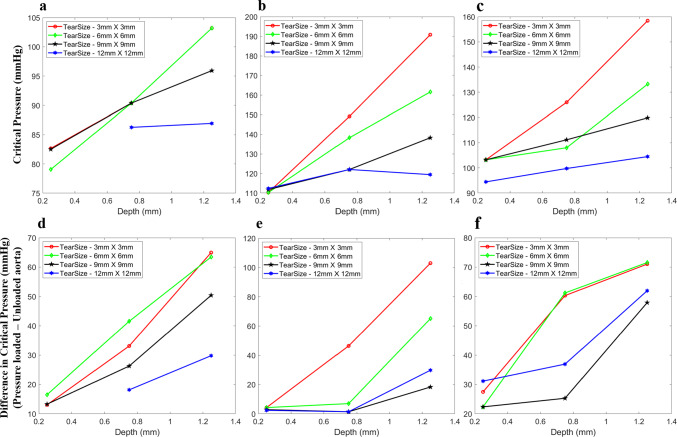
Critical pressure versus depth for the tears located at the ascending (**a**), aortic arch (**b**) and descending parts (**c**) of the unloaded aorta sample-1 obtained directly from the CT scan. The difference in critical pressures (pressure-loaded - unloaded aorta) for the tears located at the ascending aorta (**d**), aortic arch (**e**) and descending aorta (**f**) of aorta sample-1 denoting the influence of initial stresses due to diastolic pressure. Note: The 12 mm $\times$ 12 mm tear in the unpressurised aorta, close to the true lumen, caused problems in the XFEM simulation due to a lack of support from the pressure-induced stresses. Hence, a conclusive result was not attained, whereas when the aorta was pressurised, the tear propagation was simulated without issues

The diameter of the ascending aorta was greater than that of the descending aorta, significantly affecting the critical pressure at these two positions. The critical pressure versus depth for the $\theta z$ tears located at the ascending part, aortic arch and descending parts of the unloaded first aorta is shown in Figure 8b–c. It was found that the loading due to diastolic blood pressure increases the strength of the aorta; hence, the critical pressure to cause further damage increases. The critical pressures to cause further propagation for the $\theta z$ tears defined in the unloaded aorta were lower than those for the same sized $\theta z$ tears defined in the pressurised (diastolic pressure) aorta geometry. The difference in critical pressures between the unloaded aorta geometry and the pressure-loaded aorta is shown in Fig. 8d–f. Hence, including the stresses due to diastolic pressure loading clearly affects the tear initiation and propagation.

### Influence of the tear depth and size inside the medial layer on critical pressure

**Fig. 9:**
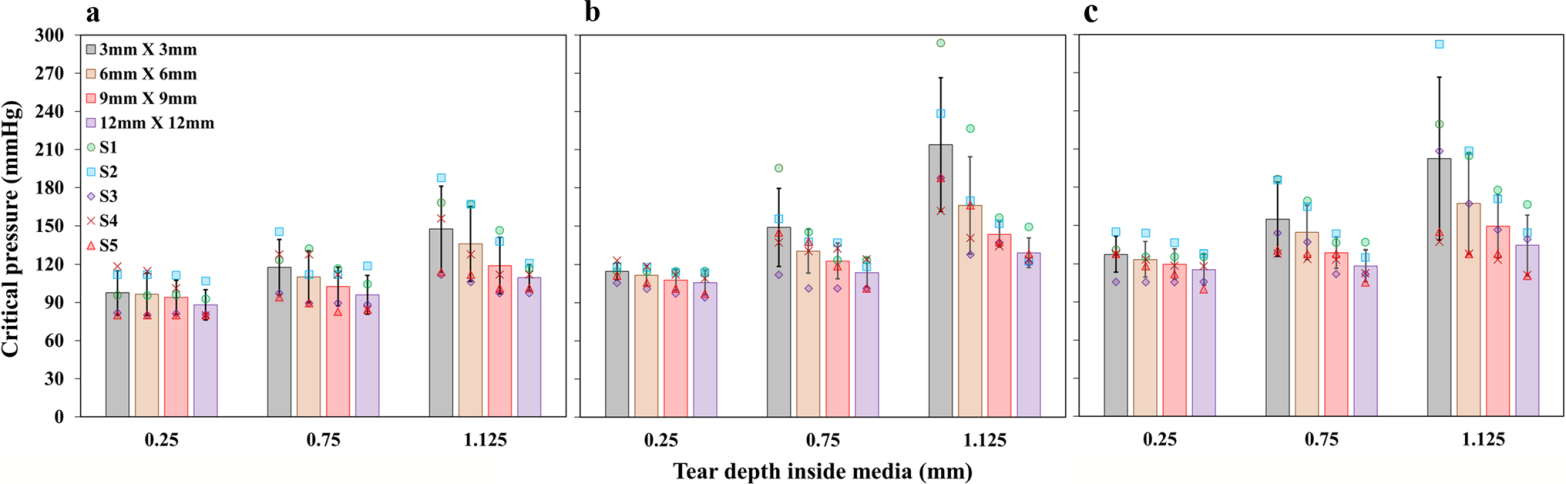
Influence of the tear size on critical pressure. Critical pressure versus depth for simultaneous pressurisation of true and false ($\theta z$ orientation) lumens. Tear sizes range from 3 to 12 mm in both $\theta$ and *z* directions. Plots for tears at ascending (**a**), arch (**b**) and descending parts (**c**) of the five-sample pressure-loaded aortas (S1–S5) for four sizes ($3 \times 3$ to $12 \times 12$ mm). Error bars denote the standard deviation

In the loaded aorta geometries, for the $\theta z$ tears, the tear sizes were kept constant, ranging from 3 to 12 mm in both directions and the corresponding critical pressures are plotted in Fig. 9. Keeping the depth fixed, which was closer to the true lumen ($d_1$=0.25 mm), the change in critical pressure with an increase in tear size was less significant for most of the aortas used in the study (Fig. 9). However, in a few cases, the difference was substantial for the tears present in the descending part (Fig. 9c) of the second aorta (17 mmHg), the arch region (Fig 9b) of the third aorta (10 mmHg), at all locations (Fig. 9a–c) in the fourth aorta (>10 mmHg) and in the aortic arch and the descending parts (>10 mmHg) of the fifth aorta (Fig. 9b and c). Otherwise, the difference in critical pressure with an increase in tear size was less than 10 mmHg for most of the tears present closer to the true lumen ($d_1$). Then, the critical pressure decreases with an increase in the tear sizes for the depths at the middle of the media ($d_2$=0.75 mm) and closer to the adventitia ($d_3$=1.125 mm).

**Fig. 10:**
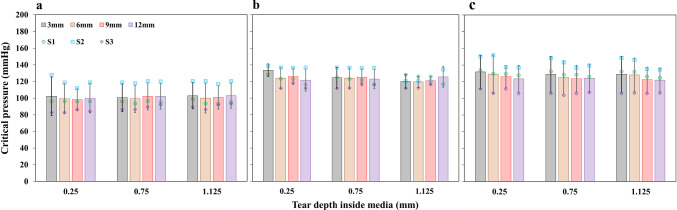
Influence of the tear location on critical pressure (tear sizes ($\theta$) were kept constant at 3 mm, 6 mm, 9 mm, and 12 mm). Critical pressure versus depth for simultaneous pressurisation of true and false ($\theta r$ orientation) lumens. Plots for tears at ascending (**a**), arch (**b**) and descending parts (**c**) of the pressure-loaded three healthy aortas (S1–S3) for four sizes (3–12 mm). Error bars denote the standard deviation

For the $\theta z$ tears, regardless of tear size, critical pressure tends to increase when the depth of the tear is increased from closer to the true lumen to the centre of the medial layer and then closer to the media–adventitia interface (Fig. 9). The maximum critical pressures were found in tears closer to the media–adventitia interface. The overall trend in the increase in critical pressure with the increase in depth inside the medial layer remains the same. But for the $\theta r$ tears defined in the first three healthy aorta samples (Fig 10), the critical pressure tends to remain almost constant (< 10 mmHg) irrespective of the increase in depth. The critical pressures for all the depths and tear sizes at the ascending aorta region was in the range of 100 mmHg (Fig. 10a), and it was around 125 mmHg for the arch and descending aorta regions (Fig. 10b and c). Also, the size of the $\theta r$ tears did not induce any change in critical pressure, unlike the depth. The critical pressure remained constant even when the tear size increased.

The tear depth inside the medial layer and the tear size significantly affects the critical pressure depending upon the orientations of the tears. The critical pressure reduced with an increase in tear size when the $\theta z$ tears were much deeper into the medial layer. However, for all the $\theta z$ orientation tears located at the depth closer to the true lumen in the ascending aorta, aortic arch and descending aorta regions, the critical pressure to cause further propagation was almost the same overall. When the tears were much deeper into the medial layer, away from the true lumen at the media centre or closer to the media and adventitia interface, it was seen that tear size has a significant effect on the critical pressure. In particular, the critical pressure of deeper tears reduced with increased tear size. When the $\theta z$ tears were closer to the true lumen, they propagated with almost the same critical pressure irrespective of the size. However, the critical pressure remains nearly constant with an increase in depth and size for the $\theta r$ tears present at all three locations, unlike in $\theta z$ tears. Thus, less deep $\theta z$ tears and all $\theta r$ tears were able to propagate further at lower critical pressures.

### Influence of the tear locations on critical pressure

The critical pressures responsible for further tear propagation when tear sizes were kept constant while the locations were varied are shown in Fig. 9 for the $\theta z$ tears defined in all the aorta samples and $\theta r$ tears defined in the three healthy aorta samples are shown in Fig. 10. For the $\theta z$ tears, other than the influence of the tear depth, the critical pressure for the tears located at the descending aorta was slightly higher than those at the aortic arch and significantly higher than the tears at the ascending aorta. In other words, the effect of the aorta diameter at the centre of the tears substantially affects the critical pressure when the tear orientation is $\theta z$.

For the $\theta r$ tears defined in the healthy aortas, the critical pressure to cause further propagation was significantly higher when the location was in the descending aorta region and lower when the area was in the ascending aorta region. The difference in critical pressure between the tears at the aortic arch and the descending aorta was insignificant. This effect of diameter on the critical pressure was the same regardless of the orientation of the tear. So, the tears at the ascending aorta region have lower critical pressures and are most likely to propagate easily. In contrast, the tears in the descending aorta regions have high critical pressure and are slightly less likely to propagate than those at the ascending aorta.

## Discussion

We have studied the influence of tear size, location and depth inside the medial layer on the onset of further tear propagation in three-dimensional aorta geometries. It was found that the deeper the tear from the true lumen, the higher the critical pressure required to cause further tear propagation for the $\theta z$ orientation tears, whereas tear depth does not influence $\theta r$ oriented tears. Brunet et al. (2023) conducted tests on dissection propagation in rabbit aortas and found that the deeper the notch depth (tear with depth d and an initial *z* length), the lower the critical pressure required to cause further propagation. They also reported that the critical pressure decreases with an increase in the notch width ($\theta$ in our study) and dissection length (*z* in our study). The same trend of decrease in critical pressure with the increase in $\theta z$ tear size was found in our studies. However, in our study, the tear depth and size did not influence the critical pressure of the $\theta r$ tears, and we found critical pressure increases with an increase in depth for $\theta z$ tears. We suspect that the initial incision notches in the Brunet et al. (2023) have both depth and some initial length. This combination (thick cut) of initial length and depth may influence the critical pressure while pressurising, however our tears were like a thin and sharp planar cut (Fig. 3a).

The differences in critical pressures influenced by the size and depth of $\theta z$ tears and the locations of both $\theta z$ and $\theta r$ tears were more significant than the influence of depth and size of the $\theta r$ tears. The former ranges more than 50 mmHg, and the latter ranges less than 5 mmHg for most cases and less than 10 mmHg for all cases. The reported critical pressures in Brunet et al. (2023) range from 200 to 1000 mmHg. However, the critical pressures in our study range from 80 to 300 mmHg. Another key finding in our study was that the tears at the ascending aorta regions always propagate with less critical pressure than those at the descending aorta regions; hence, the aorta diameters were likely to affect the critical pressure significantly. Since the rabbit aortas are smaller in diameter compared to the human aortas, we hypothesize that higher critical pressures are needed for further propagation, so future experimental studies on the healthy and dissected human thoracic aortas could add more insight into this phenomenon. Guo et al. (2019) conducted experimental research on dissection in porcine thoracic descending aortas and concluded that deeper tears propagate much further along the aorta’s axial (*z*) direction than the tears closer to the true lumen. Since the difference in critical pressure for the $\theta r$ tears with depth is significantly less, these tears could easily propagate along the radial direction along the aorta wall thickness. Consequently, we hypothesize that when these initial tears at different depths start propagating in the longitudinal direction along the aorta centreline, the shallower tears can propagate back to the true lumen and create a new entry tear, whereas deeper tears will have more strength due to the support from both the medial layer between the tear and true lumen on one side and the adventitial layer on the other. Hence, our study suggests that when the deeper tears start increasing in size, they can propagate at significantly lower critical pressures and are more likely to propagate along the axial direction than those closer to the true lumen, which might re-enter the true lumen.

Wang et al. (2018) modelled pressure-driven tear propagation in an axisymmetric geometry. The authors concluded that critical pressures to cause further propagation monotonically increase for the shorter tears ($\theta < 20^\circ$) and reduce monotonically for larger tears ($\theta \ge 60^\circ$), with the increase in depth and hypothesise that propagation direction influences this phenomenon. However, our $\theta z$ tears were mostly (75%) in the range of $13^\circ$ to $55^\circ$ and mostly propagated in the axial direction. The collagen fibres are closely aligned in the circumferential direction (Gasser et al. 2006), and tear propagation in the axial direction requires a greater critical pressure to cause damage across the collagen fibres, compared to the circumferential propagation in which the tear can propagate in between the collagen fibres more easily. Consequently, shallower tears may re-enter the true lumen easily. However, deeper tears need higher critical pressures to propagate along the radial or axial directions. Brunet et al. (2021) state that larger and deeper tears induce high-stress concentrations near the tear edges, reducing the critical pressure needed to initiate the propagation and increasing the aortic dissection risk. In an experimental study of injecting saline into the media of porcine aortas creating blebs (Tam et al. 1998) and a computational analysis of the tears of $\theta z$ configurations in a 3D cylinder (Brunet et al. 2021; Han et al. 2023), the authors state that a lower critical pressure is needed to cause further propagation for the deeper tears. Our results agree with the relationship between tear size and critical pressure, as reported in all the previous literature, but not the relationship between tear depth and critical pressure.

## Limitations

The goal of this research is to reconstruct the stress and strain fields from CT scans of individual aortas to estimate the likelihood of dissection. In general, the images show signs of arterial disease and are from older individuals, and while residual stresses and axial stretching are important features of the structure of the large arteries, the major contribution to the stress field in the vessel wall is due to the blood pressure generated by the contraction of the left ventricle of the heart, and this is the focus of our study into the onset of dissection. In doing this, we have made a number of simplifying assumptions. First, we have neglected axial prestretch since it is known to decline with age (Horný et al. 2017) and, e.g. in the human abdominal aorta approaches zero in patients > 50 years’ old. Moreover, it is challenging to implement axial prestretch in realistic human geometries based on imaging, unlike uniform cylinders. For similar reasons, residual stress is not included either, and we have not considered changes to axial prestress and aortic geometry caused by movement of the aortic root during the cardiac cycle, see e.g. Parikh et al. (2024).

A further simplification is our assumption that the wall thickness is uniform along the vessel, since clinical images only visualise the lumen, not the wall. Data on the distribution of aortic wall thickness in older patients is limited, although Rosero et al. (2011) show that mean aortic wall thickness increases with age. In our studies, we assumed constant wall thickness for the pressure-loaded configuration (the CT scan geometry), and find that the wall thickness differs by 1% to 3% between the ascending and descending aorta regions in the zero-load (zero pressure) deflated aorta geometry, from which we conclude that the alternative assumption that the wall thickness is uniform in the unloaded configuration would produce similarly small changes in the wall thickness when loaded. The Law of Laplace provides a quick calculation of the hoop or circumferential stress in a thin cylindrical wall ($T = PR/h$, where *T* is the hoop stress, *P* is the transmural pressure, *R* is the internal radius and *h* is the wall thickness), which shows that hoop stress is inversely proportional to the wall thickness in this idealised model. Sokolis (2019) conducted experimental studies on harvested porcine aortas and reported the difference in wall thickness between the ascending and descending aortas. They reported a relatively uniform diameter-to-wall thickness ratio for strips cut from both the ascending and descending aorta (unloaded) regions. The strips were not subject to residual stress or axial prestretch. While this is a significant study, these animals were young and did not have the changes that are found in older human aortas. Our results will be sensitive to wall thickness and should be revisited when further data becomes available. We note that the walls of large arteries have a complicated structure not described in the Law of Laplace above, with collagen fibres wound around the wall in two helical families. Thus the fibres contribute to the radial components of stress within the wall of the aorta to resist bulging, as shown in our simple model (Wang et al. 2015). The simple  model also demonstrated that connective tissue surrounding the wall may lead to arrest of dissection propagation. This notion is consistent with clinical observations of established dissections, which eventually propagate further as arterial tissue degrades.

Brunet et al. (2021), Han et al. (2023), Li et al. (2019), Qi et al. (2015) and Wang et al. (2017) studied the influence of axial prestretch and opening angle-induced residual stresses and fibre angles on the widening of the false lumen in a cylindrical tube. Some understanding of the likely effects of the simplifications in our study is provided by Li et al. (2019), who studied the incremental deformation analysis of arterial dissection in a cylindrical geometry for a single-layer wall using the HGO strain-energy function. Li et al. (2019) demonstrated that, when subject to residual stress and axial prestretch, a dissection is widened (opened) by increasing the blood pressure within the lumen and the dissection, decreasing the radius of the lumen, decreasing the fibre angle, decreasing the axial prestretch, and increasing the opening angle $\alpha$. The dependence on $\alpha$ implies that higher values of residual stress promote wider dissections, which is unexpected and counters the effect of increasing the axial prestretch. They also noted that displacements at the inner surface of the wall are greater than at the outer surface.

## Conclusion

From the results of our study, we conclude that

- The critical pressure for tear propagation is higher for shorter and deeper $\theta z$ tears inside three-dimensional complex aorta geometries.
- For the $\theta r$ tears, the difference in critical pressures due to the increase in depth is less and transverse propagation through the aorta wall is highly likely.
- The tears located at the ascending aorta needed less critical pressure than those at the descending aorta.

Clinically, dissection in the ascending aorta will be considered more severe than at the aortic arch and descending aorta regions due to its proximity to the aortic root. The dissection at the ascending aorta might propagate easily towards the heart, compromising the aortic root, which would be catastrophic, and our study indicates the same. The ascending aorta region is the most common site (about 50%) for aortic dissection occurrence (Khan and Nair 2002). The critical pressures increase when the tear location is moved from the ascending aorta to the descending aorta. The aorta possesses a complex geometry, containing families of fibres and smooth muscle cells, making it anisotropic. Previous numerical studies in the literature only considered simple strip geometries or simple cylindrical geometries modelled with isotropic and anisotropic material models. Hence, this study helps understand aortic dissection initiation propagation in complex three-dimensional human aorta geometries modelled with the anisotropic hyperelastic material model. The results from this study clearly show the influence of tear location, size and depth on critical pressure for dissection propagation. This knowledge has the potential to be very useful in future diagnosis and in determining the most appropriate treatment options specific to the patients faster, as aortic dissection is a very time-sensitive condition.
